# Performance of Encounternet Tags: Field Tests of Miniaturized Proximity Loggers for Use on Small Birds

**DOI:** 10.1371/journal.pone.0137242

**Published:** 2015-09-08

**Authors:** Iris I. Levin, David M. Zonana, John M. Burt, Rebecca J. Safran

**Affiliations:** 1 Department of Ecology and Evolutionary Biology, University of Colorado, Boulder, Colorado, United States of America; 2 Encounternet LLC, Portland, Oregon, United States of America; 3 Department of Electrical Engineering, University of Washington, Seattle, Washington, United States of America; Cornell University, UNITED STATES

## Abstract

Proximity logging is a new tool for understanding social behavior as it allows for accurate quantification of social networks. We report results from field calibration and deployment tests of miniaturized proximity tags (Encounternet), digital transceivers that log encounters between tagged individuals. We examined radio signal behavior in relation to tag attachment (tag, tag on bird, tag on saline-filled balloon) to understand how radio signal strength is affected by the tag mounting technique used for calibration tests. We investigated inter-tag and inter-receiver station variability, and in each calibration test we accounted for the effects of antennae orientation. Additionally, we used data from a live deployment on breeding barn swallows (*Hirundo rustica erythrogaster*) to analyze the quality of the logs, including reciprocal agreement in dyadic logs. We evaluated the impact (in terms of mass changes) of tag attachment on the birds. We were able to statistically distinguish between RSSI values associated with different close-proximity (<5m) tag-tag distances regardless of antennae orientation. Inter-tag variability was low, but we did find significant inter-receiver station variability. Reciprocal agreement of dyadic logs was high and social networks were constructed from proximity tag logs based on two different RSSI thresholds. There was no evidence of significant mass loss in the time birds were wearing tags. We conclude that proximity loggers are accurate and effective for quantifying social behavior. However, because RSSI and distance cannot be perfectly resolved, data from proximity loggers are most appropriate for comparing networks based on specific RSSI thresholds. The Encounternet system is flexible and customizable, and tags are now light enough for use on small animals (<50g).

## Introduction

Accurately quantifying interactions between individual animals has been both a long-standing interest and challenge for behavioral ecologists and wildlife epidemiologists. Social network analysis is a recent analytical approach that attempts to link individual behavior to population-level patterns and processes [1,2]. Social network analysis relies on the quantification of interactions between individuals within populations of associated animals. Proximity logging tags and collars are a recent advance that allow for automated collection of large amounts of contact rate data that can be used to construct social networks [3, 4]. Proximity loggers function as transceivers that transmit unique identification (ID) codes while simultaneously “listening” for ID codes from other nearby loggers. Upon detecting other tags, interaction data are stored directly on the proximity loggers. Programming basic aspects of these tags is fairly flexible. For example, users can set a variety of logging parameters including radio signal strength thresholds for recording contacts among tagged individuals and daily on/off settings. Proximity loggers have been successfully employed to record interactions in several systems including investigations of tuberculosis transmission in European badgers [5], social affiliation in cattle [6], reproductive behavior of island foxes [7], terrestrial social interactions in Galapagos sea lions [8], and seasonal variation in sociality and implications for infectious tumor transmission in Tasmanian devils [9].

Most studies of social interactions via proximity loggers involve mammals, and nearly all of those use larger animals (e.g., raccoon, badger, elk) to accommodate the heavy (500+g) proximity collars made by Lotek Wireless Inc. (Newmarket, ON, Canada), formerly manufactured by Sirtrack Ltd. (Hawkes Bay, New Zealand). The size of these collars depends largely on the components necessary for transceiver capability as well as substantial battery power to provide many months of data on individual contacts between collared individuals [10, 11]. Recent advances in proximity tag technology (Encounternet Ltd., Portland, OR) have led to the development of smaller proximity loggers for sea lions (65–70g; [8]), New Caledonian crows (9.5g; [12, 13]), and small passerine birds such as barn swallows (1.3g; described here). Although small (<1g) coded nanotags [14] and miniaturized Encounternet tags (<1g) operating in transmit-only mode (i.e., tags transmit ID codes but do not receive ID codes from nearby tags) [15, 16] have been previously employed, there are no published results on the use of miniaturized proximity loggers for sampling social networks in small animals such as passerine birds or rodents. Instead, previous studies that use miniaturized tags have inferred contacts via receiver stations (stationary radio signal receivers that passively monitor tag ID pulses) set to detect within a fixed radius (e.g., [14]). Social interactions can be inferred based on time stamps and conditional rule sets applied to the tag-receiver data [14]. While this approach has the advantage that the battery life in a transmit-only mode is typically much longer than a logger functioning as a transceiver (e.g., 7.5 days for Encounternet tags in transmit-only mode [15] vs. 21 hours in proximity-logging mode (reported here)), it restricts data collection on social contacts to areas near receiver stations. Here, we report the first field calibration and live animal tests of miniaturized proximity logging tags.

The number of studies that use proximity loggers has increased rapidly; however, systematic testing of the equipment has not necessarily kept pace [17, 18]. The accuracy, reliability and limitation of proximity logging systems have not always been analyzed (but see [10, 13 17–19], who do test aspects of tag reliability and reciprocal agreement). Additionally, there is a knowledge gap related to ground truthing proximity logging systems for smaller animals that are more vagile and harder to observe than larger, slower-moving mammals that have more commonly been studied with proximity logging systems. In order to reconstruct a robust social network from contact data, one needs to understand the effects of inter-logger variability in performance, reciprocal agreement of proximity loggers recording dyadic interactions, and correlate radio signal strength values to known distances via controlled field/laboratory tests of loggers not on animals. Additionally, proximity loggers might impact host condition and behavior—obvious considerations for well-planned studies. Our study differs from other proximity logger methods tests in several key areas. First, we report data from tag-tag and tag-receiver station calibration tests and data from live deployment. Second, we use our deployment data to analyze log quality and reliability and apply RSSI thresholds (that correspond to statistically robust distance categories from calibration testing) to generate different social networks. Finally, we perform relatively simple, easily reproducible calibration tests that produce results very similar to those obtained by extensive modeling of radio wave propagation [13].

We deployed miniaturized proximity tags (1.29 ± 0.025g) on free-living barn swallows (*Hirundo rustica erythrogaster*) (17.59 ± 0.881 g) and additionally systematically tested tags in the field but not on birds (referred to as calibration tests throughout) to answer the following questions: 1) What tag mounting system for field tests most accurately reflects data obtained from live, tagged birds? 2) Can we infer a particular tag-tag distance associated with a received signal strength indicator (RSSI) value provided by the tag’s digital radio for every received ID pulse? 3) How does inter-tag and inter-receiver station variability affect our RSSI and distance calibration? 4) What impact do the tags have on barn swallows in terms of changes in pre- and post-deployment mass? 5) In deployment trials on live animals, how often do tag pairs record dyadic interactions and what influences the probability of dyadic vs. single logs? 6) How frequently does one tag in a dyadic interaction record a broken log (two or more logs when the other tag records one)? 7) How different are networks constructed from different maximum RSSI thresholds?

## Materials and Methods

### Encounternet tag technology and settings

**Fig 1:**
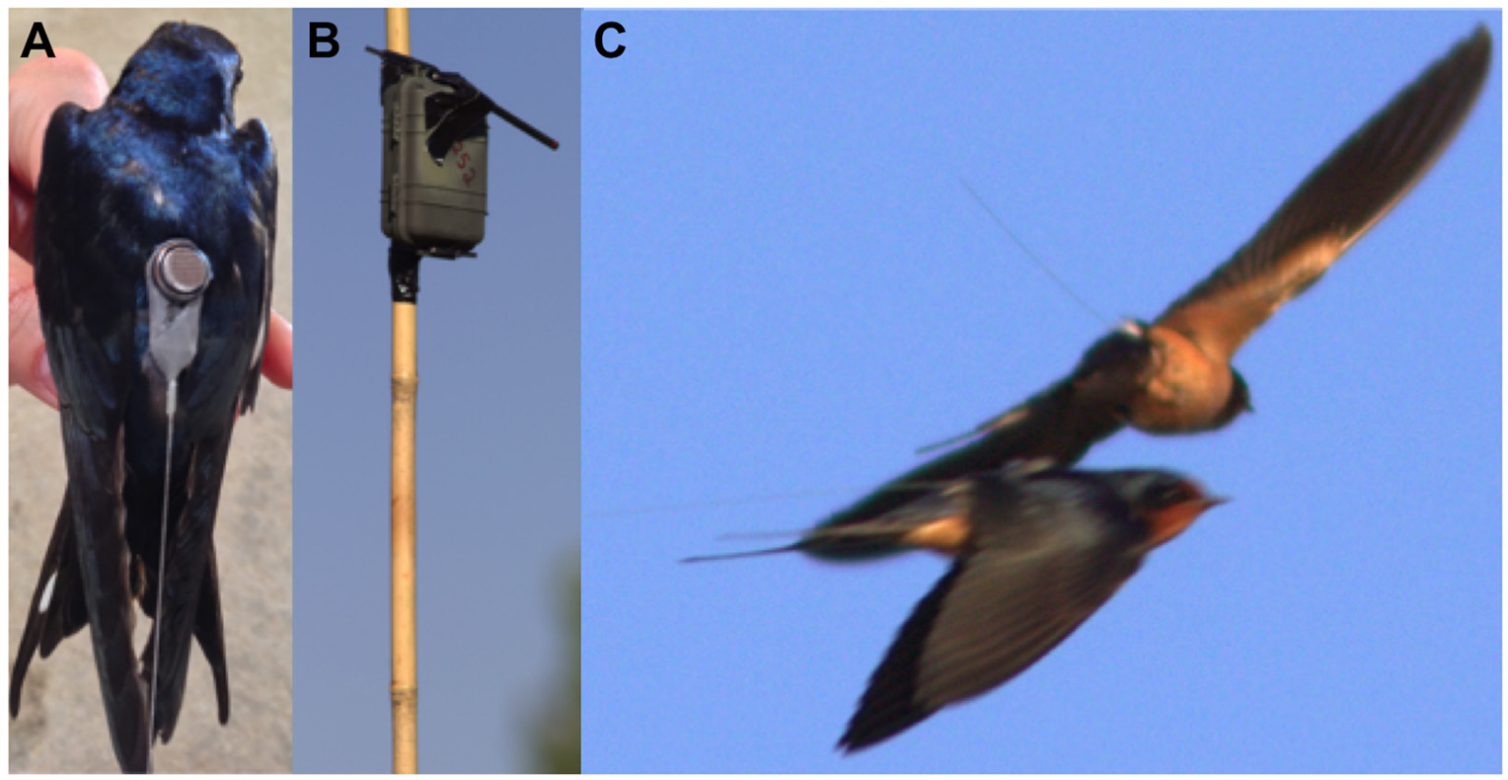
A. Barn swallow wearing an Encounternet tag. B. Encounternet receiver station. C. Tagged swallows interacting.

The Encounternet system consists of radio transceiver tags worn by animals (Fig 1A), receiver stations that monitor tag ID pulses and collect tag logs (Fig 1B), and a PC-computer running Pymaster (Encounternet software) connected to a master node device with a directional antenna used to download log data from receivers and configure tags. The UHF transceiver chip in each tag broadcasts a unique identification (ID) pulse at 433 MHz while receiving ID codes from other nearby tags. We configured the tags to only log ID pulses from nearby tags that had ≥ 0 radio signal strength indicator (RSSI) values (corresponding to a distance that was smaller than the average distance between active nests). Tags were continuously pulsing every 20 seconds and logging other tags within radio proximity between the hours of 6–9am and 5–8pm, periods of high swallow activity. Otherwise, tags were set to stay in low-power “sleep mode” with their radio turned off. Tags detected and monitored a period of close proximity (≥ 0 RSSI) to another tag as an “encounter” (Fig 1C). At the end of an encounter (when the other tag was no longer in proximity) an encounter log was saved containing a record of: the ID of the tag saving the log, the ID of the tag detected in proximity, the start date/time of the encounter, the end date/time, the minimum, mean, and maximum received ID pulse signal strength during the encounter. The tags could monitor up to 30 simultaneous encounters with different tags, and could store up to 300 encounter logs in their RAM memory. Tags were configured to record encounters up to five minutes in duration, after which the encounter was logged to memory and the tag would begin recording a new encounter (see methods on Encounternet tag deployment for how this was handled for data processing). The five-minute cap on encounter duration was intended to reduce the number of logs saved, but allow a reasonable time resolution for encounter statistics (RSSI measures, which indicate proximity). If every ID pulse received was logged, then tag memories would fill up causing logs to be lost and the number of log download events to greatly increase, reducing tag lifespan. If an encounter was less than five minutes, it will still log the actual duration. Tags were set to sleep initially, and then turn on automatically and begin logging three days after we began catching and harnessing birds to ensure we had the population tagged and that the birds were given time to settle after capture before we recorded interaction data. We placed eight receiver stations in the surrounding barn swallow habitat, which included a barn where they nest, by hanging them from the barn ceiling, mounting on 8 foot bamboo poles fixed to fences, or hanging from trees. Receiver stations passively monitor the tag pulses, which allowed us to confirm that tags were operational even if interactions were not logged. Receiver stations did not have a detection threshold set, and therefore recorded pulses from tags that corresponded to a distance of up to 100m away from the receiver station. Some tag clocks can drift as much as several seconds per day, so we used receiver stations, which had their clocks updated daily with the master device, as time synchronization sources for tags. Each receiver station transmitted its clock time every 15 minutes. If a receiver station clock had been set more recently than a tag clock, then the tag would update its clock to that of the receiver station. Additionally, the tags had a clock saver parameter activated and set to 30 seconds, which would return a tag to the correct time (within a few minutes) if the tag reset and lost its clock. The clock saver parameter works by setting the tag clock to the last time saved plus the clock saver period/2 + 2 seconds. This formula assumes the tag was reset with a two second startup delay, and with an unknown reset time since the last clock save, it sets the clock to the middle of the possible reset time range. Maintaining accurate clocks on tags helped with aligning logs between tags and ensured that all tags were active at the same time. Recorded tag ID pulses also indicate how many logs the tag has stored to memory. When tags had ≥ 10 logs stored in memory and the bird was near one of two receiver stations (in different locations in the barn) programmed to automatically collect logs, receiver stations would begin to download logs. In order to facilitate log downloading, tags turned on for 10 minutes between 11:50pm-12:00am when the birds were most likely in the barn near the receiver stations and relatively still. When tags were near the end of their anticipated battery life (based on running tags not on birds prior to deployment), remaining logs were downloaded manually using the master node device when the bird was close by (>0 RSSI, determined by manually monitoring RSSI values of the ID pulses received from the targeted tag). The protocols in this study were approved by the University of Colorado’s Institutional Animal Care and Use Committee (protocol No. 1303.02). The research was conducted on private land with permits issued by the state of Colorado’s Department of Natural Resources (Scientific Collection License No. 12TRb2005) and a Federal Bird Banding Permit (No. 23505).

### Calibration tests of proximity tags

Calibration tests of Encounternet equipment were conducted at the site of deployment (40°07’57.6”N, 105°10’40.1”W) and in a nearby field at the Dodd Reservoir Audubon site (40°06’03.6”N, 105°11’28.1”W). Testing was done in the month (August) following tag deployment on swallows; however, we would recommend doing calibration trials prior to deployment. Tests at Dodd Reservoir included comparisons of tag-tag RSSI at set distances between randomly selected tag pairs.

#### Test I: Effect of tag mount method on RSSI across variable tag-tag distances

First we tested whether tag mounting technique affected RSSI readings, as animals are known to amplify radio signals [20]. We wanted to determine what tag mounting system produced results that most closely resembled radio signal strength behavior when a tag was mounted on a bird. This would help future researchers accurately calibrate interaction distances from real deployments. We used one tag pair tested at 0, 0.1, 0.5, 1, 2, 5, 10, 20, 30, 40m to assess differences in radio signal strength amplification with three tag mount methods which were all attached to the tops of 8’ bamboo poles with the tag antenna parallel to the ground. Tags were either mounted directly on poles, mounted on thawed, dead barn swallows attached to poles, or mounted on 20g saline (0.9%) balloons made from nitrile glove fingers which were attached to the poles. For all tests, the stationary tag was set to operate in a repeater mode, where instead of sending an ID pulse, it reported the received radio signal strength from the other tag that was held at the varying distances. We recorded six RSSI readings reported by the repeater tag: three with test tag antennae parallel to one another and three with the non-stationary tag’s antenna facing away (perpendicular on the horizontal plane) from the repeater tag.

#### Test II: RSSI at variable tag-tag distances and inter-tag variability

In order to quantify how radio signal strength changed with distance as well as any inter-tag signal variability, 11 unique tag pairs were tested at distances of 0, 0.1, 0.5, 1, 2, 5 and 10m using the saline balloon mount method, which produced radio signal results that were indistinguishable from the swallow mount method. Tests were conducted using the same procedure as in test I.

#### Test III: Variability in receiver station-tag RSSI

We tested tag-receiver station RSSI for eight receiver stations tested at two distances (1m, 10m) using the same tag for all tests. In this case, the receiver station remained stationary and was set as the repeater. We recorded three RSSI values from the tag mounted on a saline-filled balloon fixed to a bamboo pole held parallel to the face of the receiver station with the receiver antenna facing up (i.e., antennae at 90°) and three RSSI values with the tag antenna pointing away from the face of the receiver.

#### Test IV: RSSI at variable tag-tag distances inside barn vs. open field

To determine the effect of environment on radio signal transmission, we repeated tag-tag RSSI vs. distance tests in the barn at the deployment site. We used the same test distances up to 10m and tested three transects in the wooden barn using one tag pair represented in the randomly paired tag-tag tests at Dodd Reservoir. One transect ran down the center of the barn aisle and another ran perpendicular to the aisle transect, passing through two horse stalls (a typical feature of barn swallow breeding sites). The third transect ran parallel to the center aisle transect, but this time against the inside wall of the barn. These tests were done exactly as described above, using tags mounted on saline balloons attached to the top of 8’ bamboo poles.

### Statistical analyses of calibration test data

We used a repeated measures ANOVA to test for differences in RSSI between tag mount types tested across the same tag-tag distances and linear mixed models to test for effects of distance, antenna orientation, individual tag effects and influence of tag pair on received signal strength indicator values. The linear mixed models, performed in R using the *lme4* [21] and *lmerTest* [22] packages, included distance, antenna orientation and the interaction between distance and antenna orientation as fixed effects and the tags and tag pair identities as random effects. We also ran an ANOVA that tested for the effects of distance (in this case distance was treated as categorical), antenna orientation and the interaction of antenna orientation and distance categories on RSSI readings between different tag pairs. Using these results, we performed a Tukey’s *post hoc* test to identify which RSSI values associated with pairs of distance categories were statistically distinguishable. This analysis would inform whether we could accurately infer proximity based on an RSSI reading from a pair of tags with reasonable confidence. We used an ANOVA to reveal any inter-receiver station variation in RSSI at the two measured distances. Finally, we used repeated measures ANOVAs to compare tag-tag RSSI (using a set tag pair) over the same test distances between the three different barn transects, and between barn RSSI readings and those obtained from the same tag pair in the field at Dodd Reservoir.

### Proximity tag deployment

We deployed 21 miniaturized proximity tags on birds (10 males and 11 females) between July 17 and 30, 2014 during second clutch initiation. This represented 85% of the total population at the site; barn swallows breed in discrete populations in human-made structures such as barns and sheds, under bridges and in culverts. Barn swallows were captured via mist nets, weighed, and fitted with a proximity tag of known mass. We attached tags to the birds using a figure-eight leg harness (e.g., [23]) made from 0.1mm nylon beading elastic. We used a harness size recommended for barn swallows [24], where the harness diameter measured 42mm when stretched between 2 small nails. Tagged individuals were monitored twice a day using the master node device to confirm that the tags were functioning properly and that the tagged animals were present near the barn during most sampling periods. We monitored tag pulses during tag active periods but always at a distance >100m from the barn in order to lessen potential impacts on bird behavior. Proximity logs (tag-tag logs and tag-receiver station logs) were periodically downloaded from eight receiver stations outside of sampling hours when tags were not actively logging. We retrieved tags between 4 and 13 days post-deployment and the birds were weighed again to calculate any change in mass during the tagging period.

### Statistical analyses of barn swallow proximity data and encounter log processing

In order to quantify impacts of tags on bird body condition, we included data from a previous tag deployment at another site one month prior, where our tag retrieval rate was higher; as the breeding season progressed, birds became harder to recapture. Thus, we could examine the change in mass post-deployment using a larger sample size (n = 21). We used t-tests to examine individual changes in mass and to test whether the sexes differed with respect to mass changes calculated post-deployment. We used a linear regression to test how mass changes varied with the time period that birds wore proximity tags. In both cases, we repeated the analyses and excluded data for two females who were noticeably gravid (carrying eggs) at the time of tagging. It is difficult to quantify effects on birds that we never managed to recapture; however, 10 of the 21 tagged birds returned to the site in 2015. Of those 10, six were never recaptured in 2014 to have their tag removed. When captured in 2015 they were not wearing a tag and we assume the tag fell off before the fall migration.

Unique tag-tag logs were identified and filtered using Encounternet’s Pymaster software. We did not impose a minimum duration threshold or require a minimum number of dyadic detections for a log to qualify as an encounter. In order to accurately quantify the number and duration of encounters, we combined logs between the same tag pair that were separated by ≤ 20 s (the tag pulse rate). Data were checked manually and logs were identified as dyads (both tags logged the encounter) or single logs recorded by one tag of the interacting pair. Independent dyads were called when encounter start and end times overlapped in encounters logged by complementary pair of tags (e.g., Tag2-Tag5, Tag5-Tag2) and dyads were separated by >20 s. Single logs detected by only one tag were also identified as independent logs if separated from another log by >20s. We tested for correspondence in the dyadic logs using paired t-tests of maximum RSSI, minimum RSSI, mean RSSI and log duration. Subsequently, a logistic regression and Wald’s tests of significance were used to determine what most influenced whether an encounter was recorded as a dyadic pair of logs by both interacting tags or as a single log recorded by one of the tags in the interacting pair. The model included maximum, minimum and mean RSSI and encounter duration. These analyses were performed on data from the first nine hours of logging. By restricting this analysis to the first nine hours we ensured that all possible dyadic data had automatically downloaded from the tags.

**Fig 2:**
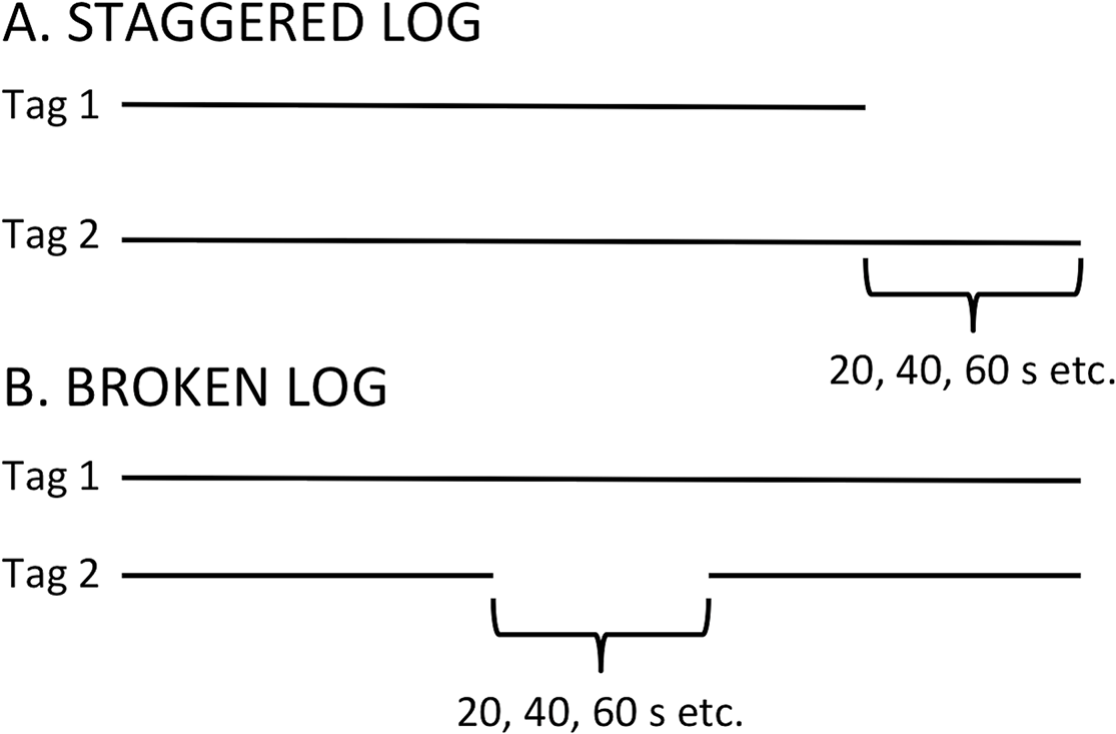
A. Illustration of a staggered log. One tag records for at least one additional pulse. In this study, the pulse rate was set at 20s. B. Illustration of a broken log. One tag records two separate logs while the other in the dyad records one continuous log.

In the case of dyadic logs, we quantified the occurrence of broken logs (logs where one tag recorded continuously while the other tag had a gap of > 21s and records two or more independent encounters) and staggered logs (logs where one tag records singly for > 21 seconds at one or both ends of a dyadic interaction)(Fig 2). We analyzed differences in RSSI values between broken and unbroken logs by pairing each broken log with an unbroken log of similar duration (average difference in paired log duration: 7.94±11.75 seconds). We used t-tests to quantify differences in log parameters (min, max, and mean RSSI, and duration) in broken and unbroken logs.

We constructed symmetrical adjacency matrices based on the total number of encounters (dyads + single logs) for two distance thresholds: one based on logs with 0+ maximum RSSI = proximity < 5m and another made from logs with 40+ maximum RSSI = proximity < 0.1m. Matrices only contained data from time periods when all tags were operational. We visualized networks in the R package *igraph* [25] with edges weighted by contact rate (# interactions) and a Fruchterman Reingold layout algorithm [26]. In order to visualize the 0+ maximum RSSI, edge weight was scaled to 1/5 so that edges were distinguishable and not obscuring nodes or other edges. All analyses were performed in R 3.1.1 (R core team 2014, http://www.R-project.org).

## Results

### Calibration tests of proximity tags

#### Test I: Effect of tag mount method on RSSI across variable tag-tag distances

RSSI readings across set distances depended on the tag mount method used for field tests. When we accounted for the variability in RSSI across given distances, we found a significant effect of mount type (repeated measures ANOVA, test type: F_2,60_ = 7.39, η^2^ = 0.45, p = 0.005). A paired t-test revealed that that the significant difference in RSSI values could be attributed to the difference between tags mounted on poles and tags mounted on dead swallows fixed to poles (tag-tag:tag-tag swallows: t_15,20_ = -4.32, Cohen’s *d* = 0.17, p = 0.001; tag-tag: tag-tag saline balloons: t_15,20_ = -1.66, p = 0.12; tag-tag swallows: tag-tag saline balloons: t_15,20_ = 1.46, p = 0.16; n = 20 per mount type).

#### Test II: RSSI at variable tag-tag distances and inter-tag variability

**Fig 3:**
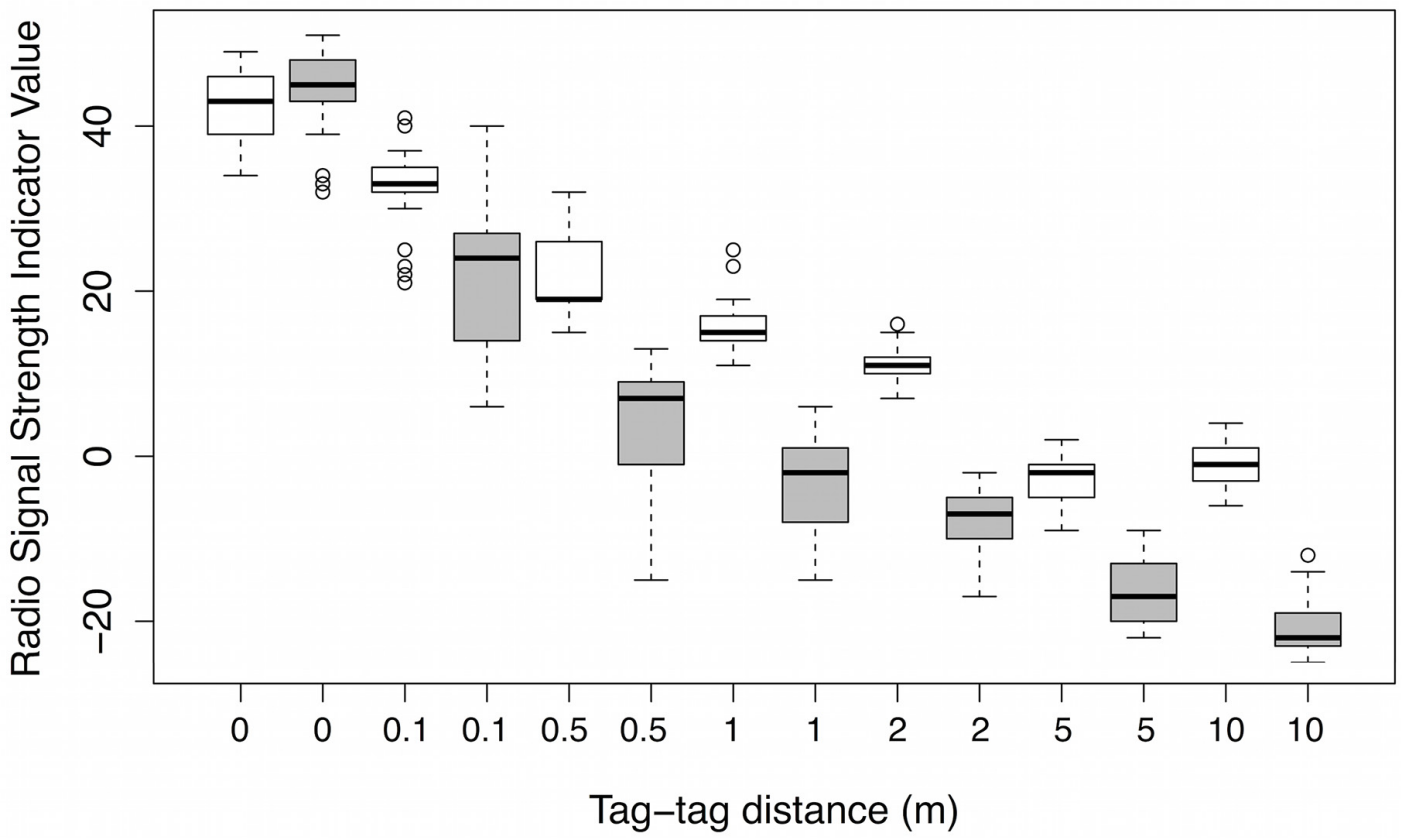
Received signal strength indicator (RSSI) values recorded between 11 tag pairs across seven different tag-tag distances. Two tag antenna orientations (parallel = white boxes, perpendicular = gray boxes) were tested for each distance and three RSSI values were recorded for each antenna orientation.

Eleven tag pairs produced consistent RSSI values across the set test distances; however, RSSI depended on several factors including distance, antenna orientation and the interaction between antenna orientation and distance (linear mixed model, fixed effects: β = -3.61, p<0.0001 for distance, β = -11.62, p<0.0001 for antenna orientation, β = -0.87, p = 0.015 for the interaction between distance and antenna orientation, with n = 462, df = 450.8 for all). RSSI values changed predictably with distance, with higher values recorded when tags were closer together (Fig 3). Antenna orientation did significantly affect RSSI readings, with higher RSSI values recorded when tag antennae were parallel vs. perpendicular (RSSI difference at each measured distance: 0m: 1, 0.1m: 10.30, 0.5m: 17.58, 1m: 19.06, 2m: 18.76, 5m: 13.03, 10m: 19.76). As the distance between tags increased, this effect of tag antenna orientation on RSSI became more exaggerated. The random effects (identity of both tags used in each pair and the tag pair) did not significantly impact the RSSI readings (linear mixed model, random effects: n = 462, df = 1, χ^2^ = 3.30, p = 0.07 for tag1, χ^2^ = 1.11, p = 0.29 for tag2, χ^2^<0.0001, p = 1 for tag pair), indicating that inter-tag variability was low. We found consistent results when we treated distance as a categorical variable (ANOVA, distance: F_6,462_ = 1047.18, η^2^ = 0.80, p<0.0001; antenna orientation: F_1,462_ = 924.39, η^2^ = 0.12, p<0.0001; interaction between antenna orientation and distance: F_6,462_ = 37.88, η^2^ = 0.03, p<0.0001); this analysis allowed us to test which distance categories could be statistically distinguishable via a Tukey’s *post hoc* test. In only one case were two distance comparisons (5m-10m) statistically indistinguishable.

#### Test III: Variability in receiver station-tag RSSI

We tested tag-receiver station RSSI values for two different distances and found similar effects of distance and tag antenna orientation as we did with tag-tag tests; however, unlike with tags, we also found substantial variability in individual receiver stations (ANOVA, F_8,96_ = 12.06, η^2^ = 0.06, p<0.0001; coefficient of variation (CV) for receiver stations at 1m: 0.23, CV for receiver stations at 10m: 1.50). Receiver stations logged lower RSSI values from tags at 10m vs. 1m, lower RSSI values when tag antennae were held perpendicular to the receiver, and we detected a greater effect of antenna orientation at the farther distance (ANOVA, distance: F_1,96_ = 1262.57, η^2^ = 0.83, p<0.0001; antenna orientation: F_1,96_ = 63.63, η^2^ = 0.04, p<0.0001; distance-antenna orientation interaction: F_1,96_ = 30.91, η^2^ = 0.02, p<0.0001). Receiver stations recorded inconsistent RSSI values from the same tag at both 1 and 10m distances. A *post hoc* test indicated that nearly half of the pairwise comparisons (12/28) between tests using different receiver stations produced significantly different results (S1 Table).

#### Test IV: RSSI at variable tag-tag distances inside barn vs. open field

There was no effect of the location of the transect within the barn on tag-tag RSSI values taken across set distances (Repeated measures ANOVA, transect: F_2,126_ = 1.77, p = 0.21). Similarly, the same pair of tags produced statistically indistinguishable RSSI readings in the barn vs. in the field at Dodd Reservoir (Repeated measures ANOVA, site; F_1,168_ = 0.18, p = 0.69).

### Mass changes following tag deployment on barn swallows

Encounternet tags (n = 21) designed for barn swallows weighed 1.29 ± 0.025g (mean ± standard deviation). Female barn swallows (n = 11) used in this study weighed on average 17.68 ± 1.23g pre-deployment while males (n = 10) weighed 17.49 ± 0.94g (mean ± standard deviation in both cases). Tags were heavier than the recommended 5% of body mass [27] (mean ± standard deviation: 7.38 ± 0.456%), but birds wore the tags for less than two weeks. Because recaptures were higher earlier in the season, we included data on mass at the time of tag retrieval for additional swallows from a previous deployment (June 2014) that did not yield usable proximity logging data. Birds lost an average of 0.12 ± 1.64g, with-4g as the largest mass loss (tag worn for 14 days) and +3g as the greatest gain (tag worn for 6 days) during the duration of tag deployment. These mass changes were not statistically significant (paired t-test: t_18_ = -0.78, p = 0.45). The female that lost 4g was gravid at the time of tagging (as was a female that lost 2g), so this mass loss does not necessarily reflect a negative impact of the tag. A linear regression of mass loss and days tagged revealed a significant negative relationship (p = 0.05, r^2^ = 0.21), with a higher mass loss in birds that wore tags for a longer time period. However, when the two gravid females were removed from the analysis, this pattern disappeared (p = 0.22, r^2^ = 0.0073). Females and males experienced similar mass changes regardless of inclusion or exclusion of the two gravid females (Welch two sample t-tests, gravid females included: t_12.08_ = -0.57, p = 0.58, gravid females excluded: t_10.19_ = 0.41, p = 0.69).

### Behavioral observations of tagged birds

We observed birds flying well, incubating with no apparent negative effects of the antenna, feeding nestlings and fledglings, and mobbing predators. We noted very little to no rubbing from either the tag or the harness upon tag retrieval; however, birds did tend to sit tipped forward on their bellies despite the tag placement over their center of mass.

### Proximity data from barn swallow tag deployment

**Fig 4:**
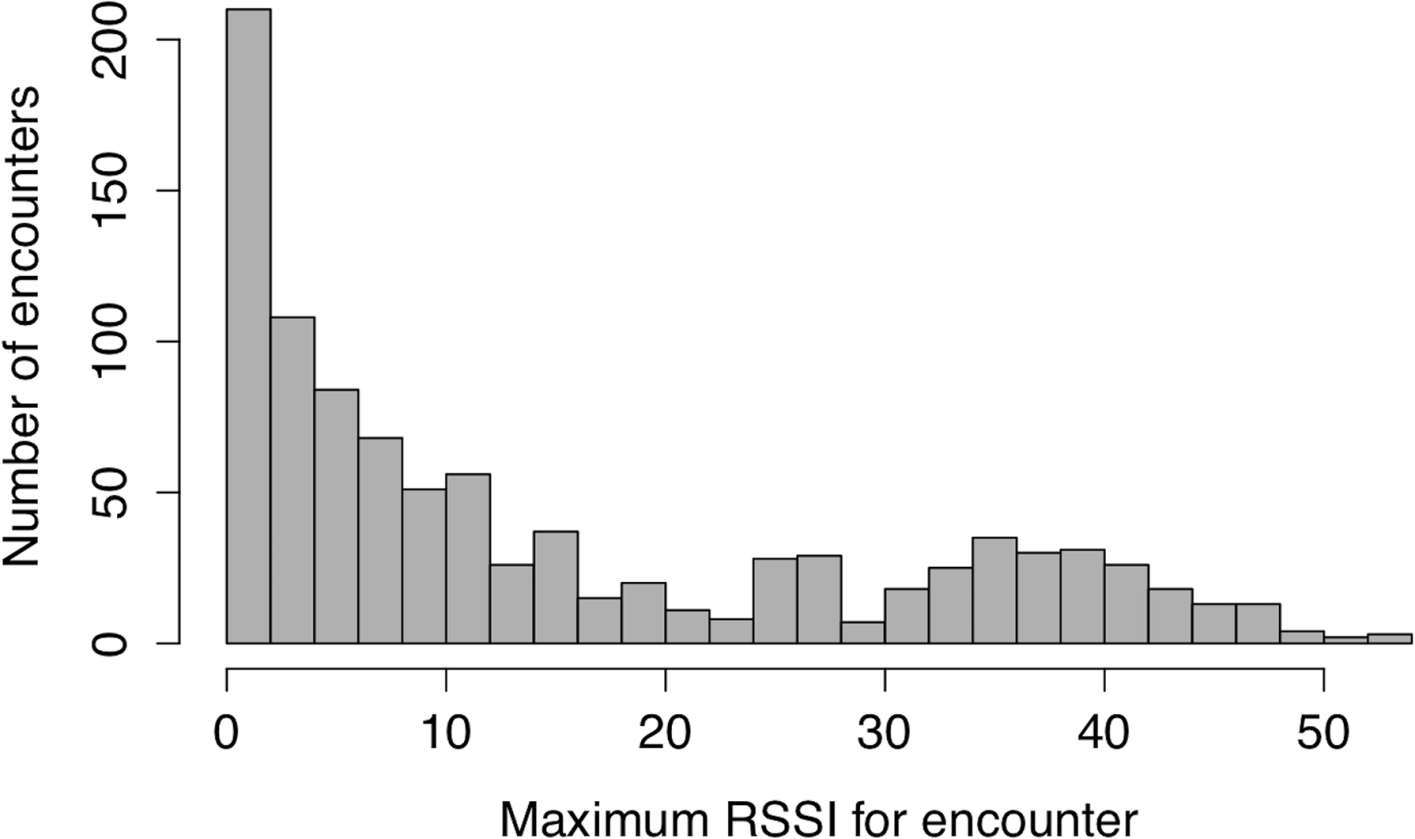
Frequencies of tag-tag logs with different maximum received signal strength indicator (RSSI) values. Tags only log encounters of maximum RSSI ≥ 0. n = 975 logs, n = 17 tagged barn swallows, and 15 hours of logging data over three days.

**Fig 5:**
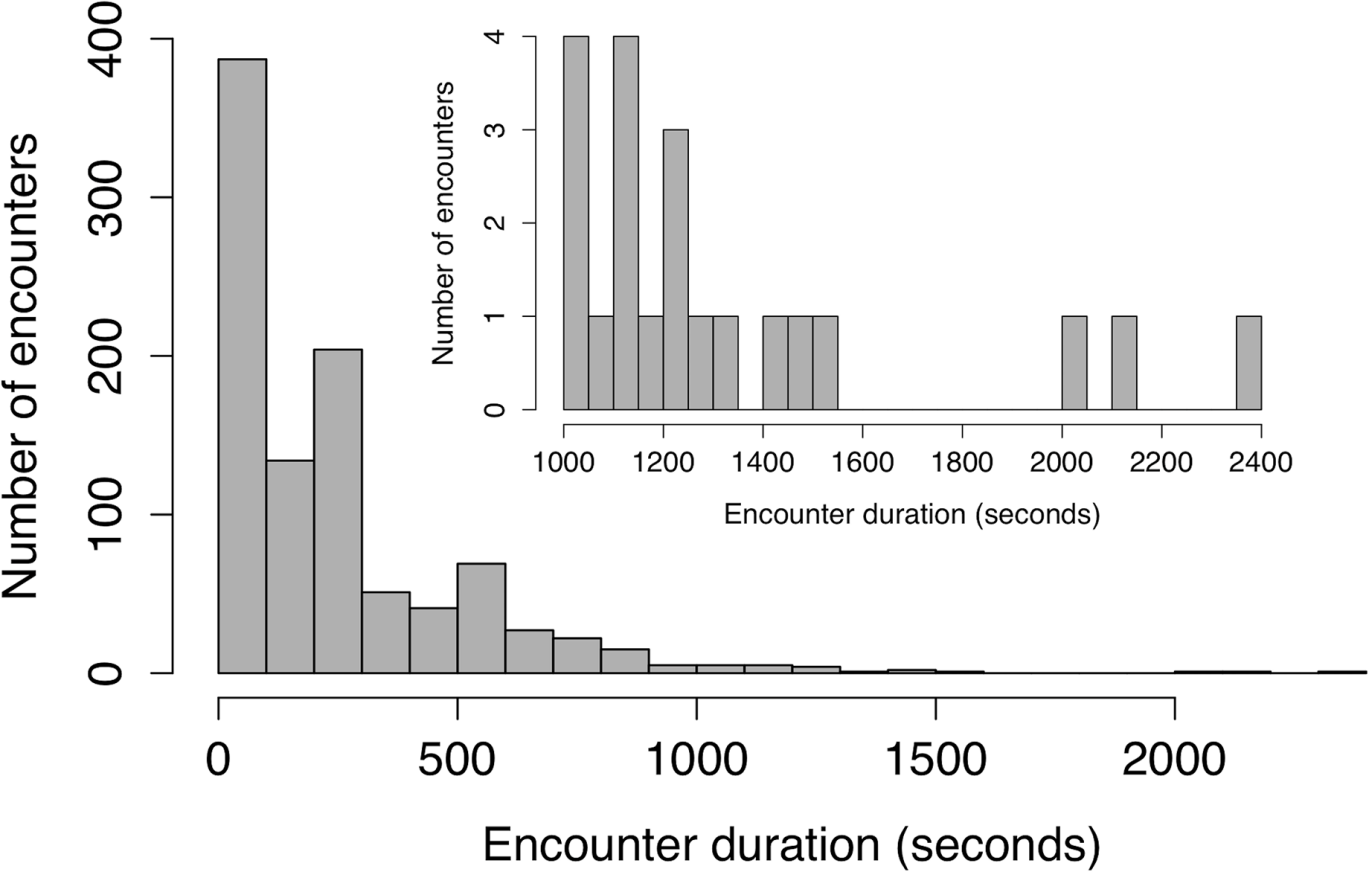
Frequencies of tag-tag durations. Inset shows the interactions >1000 s. n = 975 logs, n = 17 tagged barn swallows, and 15 hours of logging over three days.

**Fig 6:**
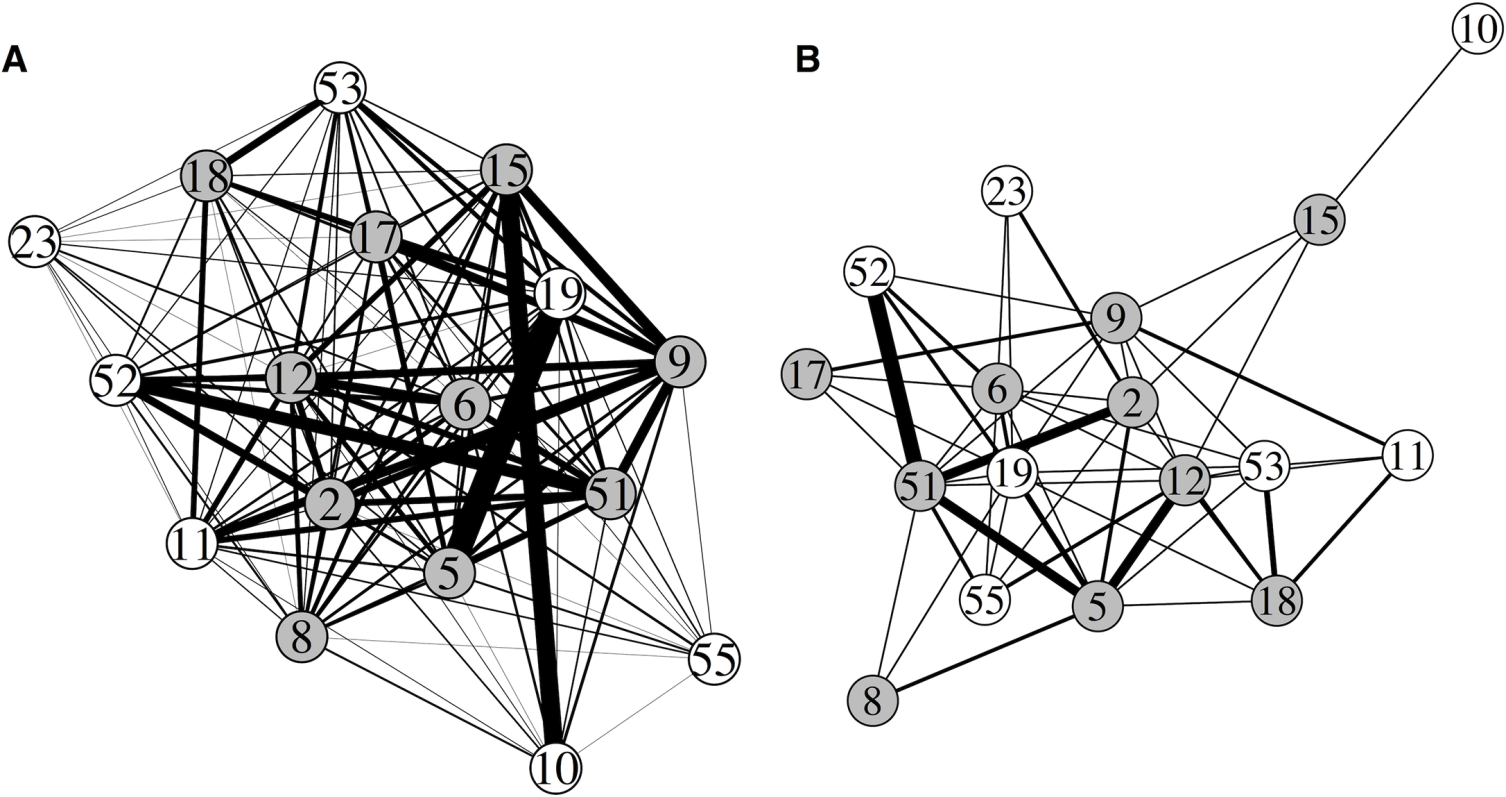
(A) Network constructed from 975 contacts between tagged barn swallows where logs had maximum RSSI values ≥ 0 = proximity < 5m (edges scaled to 1/5). (B) Network constructed from 88 contacts between tagged barn swallows where logs had maximum RSSI values ≥ 40 = proximity < 0.1m. In both cases, n = 17 tagged barn swallows with females in white and males in gray. Data are from 15 hours of logging over three days. Node labels correspond to the bird’s tag ID.

Of the 21 tags deployed on swallows, 17 tags behaved as expected given tag programing and had batteries that lasted at least five sampling periods (15 hours of logging over three days). The longest running tag lasted for 27 hours of logging (mean = 21 hours, std. dev. = 4.11, range = 15–27 hours; calculated to the nearest 3 hour sampling block). Subsequent analyses were performed using the first 15 hours of logging data when all 17 tags were functioning, unless stated otherwise. We recorded 975 tag-tag logs during this time period with a 0 maximum RSSI detection threshold (Fig 4). During this time we also recorded 27,043 tag-receiver station logs (S1 Fig); tag-receiver station logs do not have a RSSI detection threshold like tag-tag logs and every ID pulse received is logged separately. We recorded no phantom tag-tag logs where a non-existent tag ID was implicated in an encounter. Tag-tag logs recorded a range of RSSI values from-17 (prior to setting tag-tag detection threshold to 0 RSSI 24 hours after tag turn on) to 57. There was an exponential distribution of log durations; we recorded a large number of very brief interactions and few interactions longer than 1000 seconds (Fig 5). There were marked qualitative differences between networks made from different maximum RSSI thresholds (Fig 6). The network constructed from all contacts that had positive maximum RSSI values included 975 interactions between the 17 tagged individuals compared to 88 contacts when a 40 RSSI threshold was imposed.

### Analyses of tag-tag log reliability

**Fig 7:**
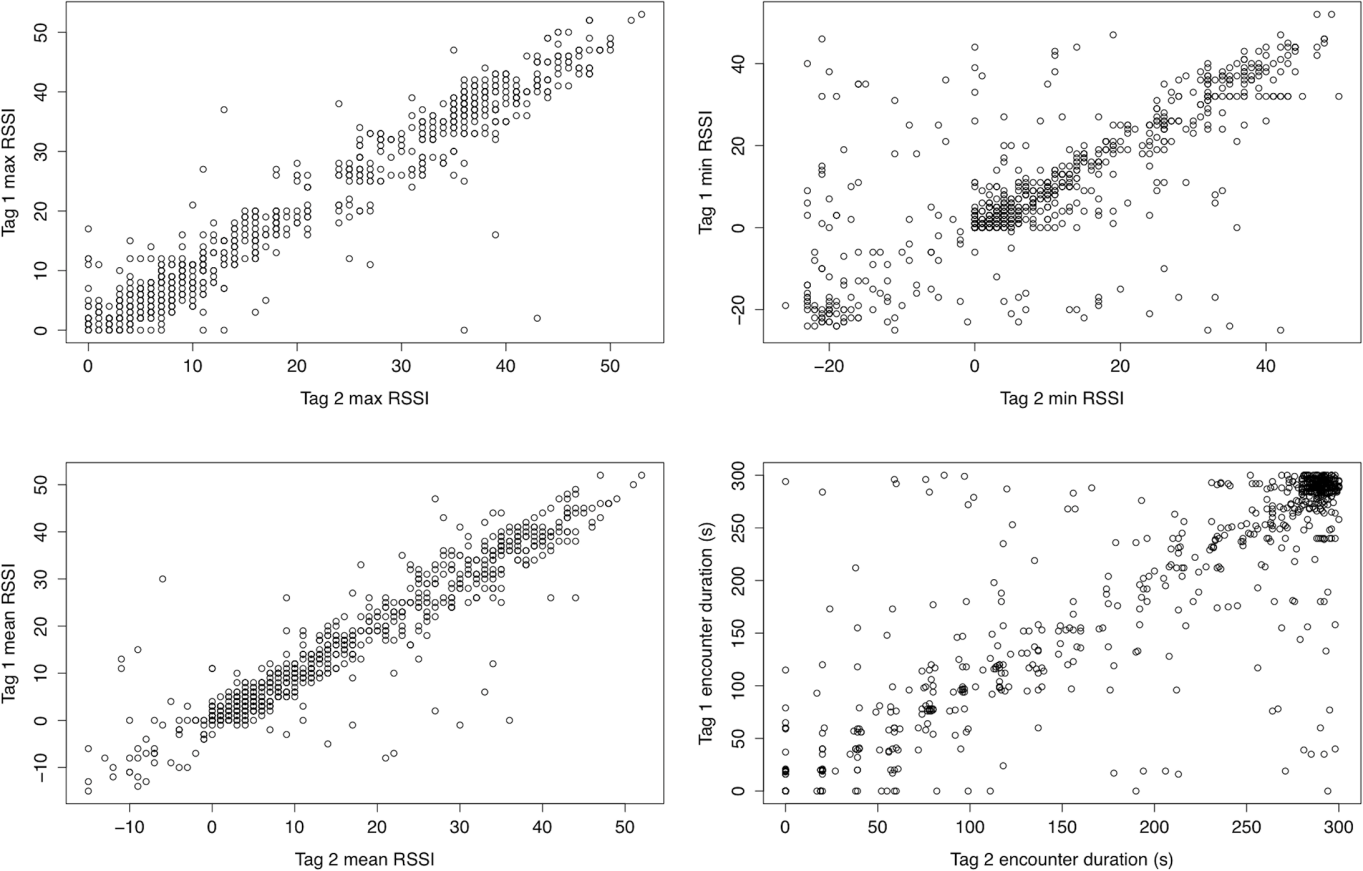
Reciprocal agreement in logs from all tags recording dyadic interactions. RSSI = received signal strength indicator values. n = 733 logs from n = 17 tagged barn swallows from all recorded logs over five days.

We captured 975 logs during deployment, 356 (36.5%) of which were dyadic with the remaining 619 (63.5%) logged only by one of the interacting tags. The probability of both tags recording an encounter (based on encounters ≤ 5 min) depended on the minimum RSSI for the encounter and the encounter duration (Wald’s test on logistic regression results, minimum RSSI: χ^2^ = 11.6, df = 1, p<0.0006; duration: χ^2^ = 31.3, df = 1, p<0.0001). Encounters were more likely to be dyadic if the encounter included higher minimum RSSI values (indicating a closer minimum distance between logging tags during the encounter). Additionally, encounters were more likely to be dyadic if they were longer in duration. We calculated mean differences between all dyadic logs (≤ 5 min) recorded during the deployment (n = 733) using the absolute value of differences in maximum, minimum and mean RSSI as well as encounter duration between data collected by both tags in each dyadic encounter (Table 1). Paired t-tests on dyadic data revealed strong correspondence between data collected by each tag, especially for maximum and mean RSSI (Fig 7) (maximum RSSI: t_732_ = -0.74, p = 0.46; minimum RSSI: t_732_ = 0.70, p = 0.48; mean RSSI: t_732_ = -0.64, p = 0.52; duration: t_732_ = -1.06, p = 0.29). We recorded a total of 37 broken logs and 15 staggered logs. Broken logs had significantly lower average maximum RSSI values (Broken logs average max RSSI: 18.51, unbroken log average max RSSI: 26.90; t-test: t_31,32_ = -2.86, Cohen’s *d* = 0.63, p = 0.007), lower average minimum RSSI values (Broken logs average min RSSI: 6.76, unbroken log average min RSSI: 16.52; t-test: t_31,32_ = -3.34, Cohen’s *d* = 0.77, p = 0.002), and lower average mean RSSI (Broken logs average mean RSSI: 14.65, unbroken log average mean RSSI: 22.67; t-test: t_31,32_ = -6.14, Cohen’s *d* = 0.71, p<0.0001). Average RSSI spread (RSSI max-RSSI min) did not differ between broken and unbroken logs (Broken logs average RSSI spread: 24.59, unbroken log average RSSI spread: 25; t-test: t_31,32_ = -0.09, p = 0.93). Thus, broken logs occurred in instances where tagged birds were farther apart rather than in instances where interactions included a lot of variation in RSSI values, indicating considerable movement during the interaction.

**Table 1:** Differences in maximum, minimum and mean RSSI and duration logged by both tags for all dyadic pairs.

Dyadic log measure	Mean difference	Standard deviation of difference	Range of difference
Maximum RSSI	2.74	3.21	0–41
Minimum RSSI	6.26	10.40	0–67
Mean RSSI	3.03	4.08	0–36
Duration (s)	26.76	45.91	0–294

## Discussion

Overall, Encounternet tags performed well in calibration tests and in deployment on live barn swallows. We were able to statistically distinguish between RSSI values at different distance categories despite the radio signal strength variation introduced by antennae orientation. We did not find an effect of individual tag or tag pair on RSSI values at different distances indicating low within-tag variability in radio signal strength. However, we did find that receiver stations were more variable in their received radio signal strength from tags. We collected 975 proximity logs from the live deployment. Although approximately two-thirds of the logs from tags on swallows were not dyadic, these logs tended to be shorter duration logs and logs with low minimum RSSI values indicating animals that were farther away during the encounter.

Despite being over the 5% of body mass recommendation for tag attachment [27], barn swallows appeared to behave normally throughout the field test. The birds showed a decrease in body mass, but they lost on average less than 1% of their body mass per day. As the time of deployment was kept relatively short, we do not expect a negative impact on the survival of the birds. It is important to keep in mind that logger mass relative to animal body mass will be experienced differently depending on how long the logger is worn and the activities of the animal during the logging duration. Researchers routinely tag animals with loggers close to 5% of body mass during energetically expensive life stages such as migration [28]. Additionally, it is difficult to determine whether this 5% rule does represent a critical tipping point for animal welfare as few studies investigate effects of heavier loggers on animal behavior and survival [28]. Our loggers were worn for a shorter duration than many telemetry studies, especially those where birds wear geolocators for over half a year [29, 30]

We obtained reliable data from the field test; however, many (63.5%) of the tag-tag logs were only recorded by one tag rather than as a dyad. This is similar to other studies using proximity loggers; for example, Meise et al. [8] report 38.5% of all logs as single interactions. How these single logs are filtered varies widely between studies. In the only other avian study that used Encounternet tags in proximity logging mode, adjacency matrices were calculated using the larger number of interactions logged by either member of each dyad [12]. If we had used this strategy, we would have grossly underestimated the interactions between tagged individuals. Many studies also discard all interactions ≤ 1 second long [11, 18] or those logged as zero seconds in the case of another Encounternet study [8]. Authors argue that these brief interactions are tagged animals barely coming in detection range of one another [8]. While this might be true for some of our 212 interactions with zero second durations (corresponding to one tag pulse, indicating that the encounter could have a maximum duration of 39 seconds due to our 20 second pulse rate), we have logs, recorded both from single tags and as dyads, that were recorded with a duration of zero but have a large positive RSSI maximum, indicating a close proximity interaction (18 logs with duration 0 >20 RSSI; 3 logs with duration 0 > 40 RSSI)(Fig 1C). This is evidence that these are real encounters that should not be discarded. However, this decision should take into consideration differences in behavior and speed between animals like sea lions or cattle on land and barn swallows in flight. For example, swallows are territorial around their nest but many interactions take place outside of their nest territories. Our tag data reflect brief—but sometimes close proximity—interactions between swallows on the wing (Fig 1C). Finally, unlike other studies [10,31], we recorded no phantom tag-tag logs where a non-existent tag ID was implicated in an encounter.

Although we found that dyadic logs tended to be longer duration logs, we also had many instances of longer duration single logs. The probability of a dyadic log also depended on the minimum RSSI value during the encounter. Single logs often included a low (negative) minimum RSSI value, indicating that the birds were far apart for a portion of the interaction. If pulse detection decreases with distance as Mennill et al. [15] have shown, and birds only spend a brief portion of the total encounter duration near each other, it might not be surprising that encounters including larger distances may not be logged as dyads. Additionally, our field tests indicated that antenna orientation—particularly at greater tag-tag distances—affected RSSI values, so one tag might have a stronger detection probability due to higher RSSI readings. Because the detection threshold for a tag-tag log was set at 0 RSSI, some single logs could be dyads where the RSSI values recorded by the other tag fell below that threshold due to antenna orientation and the data were not stored in memory as an encounter.

Thirty-seven of 356 dyadic logs (10.4%) were recorded as broken logs, with one tag recording two or more interactions while the other tag recorded continuously during that time. This is low compared to proximity collar/tag validation studies; Meise et al. [8], who use Encounternet tags, report that 42% of all encounters were recorded as multiple logs. Drewe et al. [18] report 15% broken logs from proximity collars on cattle. The longest interaction recorded in Drewe et al. [18] was 87s compared to our 2,378 second dyadic association. The probability of broken logs probably increases with longer encounter durations. However, one must keep in mind that our tags would log maximum of five-minute (300 seconds) interactions and we would manually combine consecutive logs that were separated by time intervals that matched the tag pulse rate. Broken logs could be a result of logs generated between moving birds where one pulse received by one tag falls below the RSSI cutoff.

When our tags did record dyadic logs, the reciprocal agreement was reasonably high, especially for the maximum and mean RSSI values recorded by both tags. Other studies report similar variation in reciprocity [8, 18] while others report worse [31]. We cannot expect perfect reciprocity, as radio waves can be reflected, refracted or absorbed by features in the environment [31]. Some of the variation in encounter duration for dyadic logs might be explained by tag clock drift. Our system was programmed to synchronize tag clocks every 15 minutes if a receiver station had more recently updated its clock than the tag. However, a tagged bird would need to be within radio range of a receiver station (<100m) to receive an update. Finally, in our field test we noted that when a tag’s battery life was nearing the end, the tag would still pulse and was detected by other tags and receivers but would not log interactions onto its own memory because it would reset and lose logs. This contributed to some of the single logs, which were more frequent on the last day of logging. Ideally, our tags would always record dyadic interactions; however, we should not throw out good data simply to achieve perfect redundancy. Because we found a logical basis for the factors influencing probability of dyadic logs (higher minimum RSSI, longer duration) and good reciprocal agreement across a range of encounter durations and encounter proximities, we are confident in our data despite numerous single tag logs.

Our systematic tests of RSSI between tag pairs and tag-receiver stations over set distances revealed little to no inter-tag variation but substantial variation between receiver stations. Inter-receiver station variability was also found by Mennill et al. [15]. We only used receiver stations to synchronize tag clocks and monitor tag battery life. Systems that rely on receiver stations to infer interactions from transmitter tags will need to carefully test for individual variation in receiver stations as well as the effect of variation introduced by habitat and topography and correct for any biases. We demonstrated that doing tag-tag tests using tags mounted on swallow-sized saline balloons produces RSSI values that more closely resembled results obtained with tags mounted on dead swallows. Radio wave absorption by the animal alters (and in our case seemed to amplify) the radio signal. Other researchers have used similar approaches to approximate the effect of the animal (laboratory tests using collars on 2 liter plastic bottles filled with saline [18], quail skins stuffed with chicken gizzards [13]). We recommend that future field tests of proximity loggers be conducted using similar methods to do the best possible job of converting RSSI values into robust estimates of tag-tag distance for each encounter.

Systematic tests of tag-tag RSSI values at different distances revealed that RSSI changes predictably with distance, consistent with other studies [8, 15]. The general pattern was that RSSI values became more variable when tags were farther apart. We detected significant effects of antenna orientation and an interaction between distance and antenna orientation. We expected that antenna orientation would matter, as this has been found before with Encounternet tags and proximity collars [8, 10, 15]. Additionally, we observed that antenna orientation became more influential at larger distances between tags. Despite that, we were able to statistically distinguish between RSSI values across all of the tested distances except for those recorded at 5m and 10m, regardless of antenna orientation. However, because RSSI and distance cannot be perfectly resolved, data from proximity loggers are most appropriate for comparing networks based on distinct RSSI thresholds. Rutz et al. [13] came to a similar conclusion based on their calibration tests; they highlight the utility of proximity loggers for distance-binning but caution against converting individual RSSI values into distances given the effects of antenna orientation, radio wave propagation, and habitat. From our tests, we found that 5–10m corresponds to the upper limit of distance discrimination for tag-tag logs. From the test for mount type, which included even farther tag-tag distances, we noted that RSSI values plateau around 5–10m and only slightly lower RSSI values are recorded up to 40m. Our distance calibration/antenna orientation results show improvement in terms of accuracy over other reports of antenna orientation effects; Meise et al. [8] conclude that 2m categories should be used to differentiate between different proximity encounters (but note they had to adapt antennae for underwater deployment which could impact distance-RSSI calibration). Our tag-tag RSSI-distance calibration gave us good resolution, and for our ongoing work using proximity tags in barn swallows we are most interested in close proximity encounters (e.g., data from tag-tag logs that have RSSI values ≥ 40), where our distance resolution was greatest.

We used data from the deployment to construct two different social networks based on different maximum encounter RSSI values: one that included social interactions (within 5m) and one restricted to body contact interactions (<0.1m). Quantitative analyses of these networks are the focus of future publications; however, readers can see marked differences in the number of interactions in the different networks and in the network density (proportion of possible edges). There is much discussion about what can be inferred from proximity and contact rate data (e.g., [32, 33]), and we argue, as others have [14], that these logging techniques are best applied in systems where we understand something about the context of social interaction. Barn swallows are facultatively social and interact in and around the structure they share as a breeding site. We know the phenotypic characteristics that are preferred by females [34] as well the connections between phenotype and physiology [35, 36]. This provides a strong foundation upon which we can ask how social behavior might mediate the link between sexual signals and physiology. Our preliminary data show that social networks constructed using proximity loggers have exciting connections to both phenotype and physiology (Levin et al. unpublished data).

There are still many challenges associated with research using proximity loggers. Currently, we do not get many days of data from miniaturized tags (sampling across three days in this study), even when we sample for less than ten hours per day. This is limited by battery life, and unless battery technology improves, or other components of the tag can be further miniaturized to accommodate larger batteries, this will continue to be a limiting factor. Considering that temporal network dynamics are of growing interest (e.g., [37, 38]), the short battery life of proximity tags might reduce the applicability of the system for questions related to temporal dynamics. However, scheduling capabilities are in development that will allow for tags to turn on and off according to user settings; this could significantly increase the temporal capture of interaction data. Also, the tags used in this study did not have non-volatile memory, so any glitches, usually due to low battery voltage, led to data loss (note tags begin resetting after the 15 hours of logging used for these analyses). This problem is largely resolved, because the new model of small bird tag has onboard non-volatile memory, so data are retained even when tags reset or batteries run down. Battery life also currently limits tag ID pulse rate, which is suspected to have an impact on tag-tag log reciprocal agreement. If better batteries could support a more frequent pulse rate, reciprocal agreement and the probability of dyadic logs might increase substantially. Overall, proximity logging technology has the potential to greatly increase the quality and quantity of interaction data [4, 14] and the technology is now light enough for use on small birds. Some remaining challenges are associated with processing and filtering large data sets of contact rates that accompany this unprecedented increase in interaction data.

## Supporting Information

S1 Fig Frequencies of receiver station-tag logs. Receiver stations logged any detected tag pulse regardless of received signal strength indicator (RSSI) value. n = 27,043 logs, n = 17 tagged barn swallows, and 15 hours of logging over three days.
(TIF)

S1 Table Tukey’s *post hoc* test results for tag-receiver station calibration tests using eight different receiver stations tested at two distances (1m and 10m) from the same tag. Bold values indicate significant variation in base station readings between receiver station pairs.
(DOCX)
