# Supplementary material for: Performance of Encounternet Tags: Field Tests of Miniaturized Proximity Loggers for Use on Small Birds
Source: PLoS One. 2015 Sep 8;10(9):e0137242. doi: 10.1371/journal.pone.0137242 (PMC4562698; doi:10.1371/journal.pone.0137242)
Supplement: S1 Table — Bold values indicate significant variation in base station readings between receiver station pairs. (DOCX) [file pone.0137242.s002.docx]

| Receiver station combination | Difference in RSSI reading | Lower limit | Upper limit | Adjusted p-value |
| --- | --- | --- | --- | --- |
| B-A | 1.42 | -1.80 | 4.63 | 0.87 |
| C-A | 2.58 | -0.63 | 5.80 | 0.21 |
| **D-A** | **6.42** | **3.20** | **9.63** | **0.00** |
| **E-A** | **5.00** | **1.79** | **8.21** | **0.00** |
| **F-A** | **6.83** | **3.62** | **10.05** | **0.00** |
| **G-A** | **3.58** | **0.37** | **6.80** | **0.02** |
| **H-A** | **6.33** | **3.12** | **9.55** | **0.00** |
| C-B | 1.17 | -2.05 | 4.38 | 0.95 |
| **D-B** | **5.00** | **1.79** | **8.21** | **0.00** |
| **E-B** | **3.58** | **0.37** | **6.80** | **0.02** |
| **F-B** | **5.42** | **2.20** | **8.63** | **0.00** |
| G-B | 2.17 | -1.05 | 5.38 | 0.43 |
| **H-B** | **4.92** | **1.70** | **8.13** | **0.00** |
| **D-C** | **3.83** | **0.62** | **7.05** | **0.01** |
| E-C | 2.42 | -0.80 | 5.63 | 0.29 |
| **F-C** | **4.25** | **1.04** | **7.46** | **0.00** |
| G-C | 1.00 | -2.21 | 4.21 | 0.98 |
| **H-C** | **3.75** | **0.54** | **6.96** | **0.01** |
| E-D | -1.42 | -4.63 | 1.80 | 0.87 |
| F-D | 0.42 | -2.80 | 3.63 | 1.00 |
| G-D | -2.83 | -6.05 | 0.38 | 0.13 |
| H-D | -0.08 | -3.30 | 3.13 | 1.00 |
| F-E | 1.83 | -1.38 | 5.05 | 0.64 |
| G-E | -1.42 | -4.63 | 1.80 | 0.87 |
| H-E | 1.33 | -1.88 | 4.55 | 0.90 |
| G-F | -3.25 | -6.46 | -0.04 | 0.05 |
| H-F | -0.50 | -3.71 | 2.71 | 1.00 |
| H-G | 2.75 | -0.46 | 5.96 | 0.15 |
